# Evaluation of Survival Following Surgical Resection for Small Nonfunctional Pancreatic Neuroendocrine Tumors

**DOI:** 10.1001/jamanetworkopen.2023.4096

**Published:** 2023-03-28

**Authors:** Toshitaka Sugawara, Salvador Rodriguez Franco, Michael J. Kirsch, Kathryn L. Colborn, Jun Ishida, Samuele Grandi, Mohammed H. Al-Musawi, Ana Gleisner, Marco Del Chiaro, Richard D. Schulick

**Affiliations:** 1 Division of Surgical Oncology, Department of Surgery, University of Colorado School of Medicine, Aurora; 2 Department of Hepatobiliary and Pancreatic Surgery, Graduate School of Medicine, Tokyo Medical and Dental University, Tokyo, Japan; 3 Department of Surgery, University of Colorado School of Medicine, Aurora; 4 Department of Biostatistics and Informatics, University of Colorado School of Medicine, Aurora; 5 Surgical Outcomes and Applied Research Program, University of Colorado School of Medicine, Aurora; 6 University of Colorado Cancer Center, University of Colorado School of Medicine, Aurora

## Abstract

**Importance:**

The number of patients with small nonfunctional pancreatic neuroendocrine tumors (NF-PanNETs) is increasing. However, the role of surgery for small NF-PanNETs remains unclear.

**Objective:**

To evaluate the association between surgical resection for NF-PanNETs measuring 2 cm or smaller and survival.

**Design, Setting, and Participants:**

This cohort study used data from the National Cancer Database and included patients with NF-pancreatic neuroendocrine neoplasms who were diagnosed between January 1, 2004, and December 31, 2017. Patients with small NF-PanNETs were divided into 2 groups: group 1a (tumor size, ≤1 cm) and group 1b (tumor size, 1.1-2.0 cm). Patients without information on tumor size, overall survival, and surgical resection were excluded. Data analysis was performed in June 2022.

**Exposures:**

Patients with vs without surgical resection.

**Main Outcomes and Measures:**

The primary outcome was overall survival of patients in group 1a or group 1b who underwent surgical resection compared with those who did not, which was evaluated using Kaplan-Meier estimates and multivariable Cox proportional hazards regression models. Interactions between preoperative factors and surgical resection were analyzed with a multivariable Cox proportional hazards regression model.

**Results:**

Of the 10 504 patients with localized NF-PanNETs identified, 4641 were analyzed. These patients had a mean (SD) age of 60.5 (12.7) years and included 2338 males (50.4%). The median (IQR) follow-up time was 47.1 (28.2-71.6) months. In total, 1278 patients were in group 1a and 3363 patients were in group 1b. The surgical resection rates were 82.0% in group 1a and 87.0% in group 1b. After adjustment for preoperative factors, surgical resection was associated with longer survival for patients in group 1b (hazard ratio [HR], 0.58; 95% CI, 0.42-0.80; *P* < .001) but not for patients in group 1a (HR, 0.68; 95% CI, 0.41-1.11; *P* = .12). In group 1b, interaction analysis found that age of 64 years or younger, absence of comorbidities, treatment at academic institutions, and distal pancreatic tumors were factors associated with increased survival after surgical resection.

**Conclusions and Relevance:**

Findings of this study support an association between surgical resection and increased survival in select patients with NF-PanNETs measuring 1.1 to 2.0 cm who were younger than 65 years, had no comorbidities, received treatment at academic institutions, and had tumors of the distal pancreas. Future investigations of surgical resection for small NF-PanNETs that include the Ki-67 index are warranted to validate these findings.

## Key Points

**Question:**

Is surgical resection in patients with small nonfunctional pancreatic neuroendocrine tumors (NF-PanNETs) associated with improved survival?

**Findings:**

In this cohort study of 4641 patients with NF-PanNETs, surgical resection was associated with increased survival only for patients with tumors measuring 1.1 to 2.0 cm but not those with subcentimeter tumors.

**Meaning:**

Findings of this study suggest that surgical resection for select patients with small NF-PanNETs needs to be further investigated to verify the results.

## Teaser

This cohort study evaluates the association between surgical resection and survival for patients with small, localized nonfunctional pancreatic neuroendocrine tumors.

## Introduction

Pancreatic neuroendocrine neoplasm (PanNEN) was renamed and differentiated from carcinoid tumor by the World Health Organization (WHO) in 2000.^1^ Due to greater awareness of PanNENs and the progressive development of imaging modalities, the incidence of PanNENs (including nonfunctional PanNENs [NF-PanNENs]) in the US increased between 2000 and 2016 from 0.27 to 1.00 per 100 000 cases annually.^2^ Pancreatic neuroendocrine neoplasms have varying rates of malignancy, and the WHO grading classification was revised in 2010^3^ and 2017.^4^ The WHO 2017 grading classified the tumors as well-differentiated pancreatic neuroendocrine tumors (PanNETs) and poorly differentiated pancreatic neuroendocrine carcinomas (PanNECs) based on tumor differentiation, mitotic count, and Ki-67 index.

Surgical resection has been the first-line treatment for patients with functional or symptomatic, localized, low-grade PanNETs. Meanwhile, the treatment for small (≤2 cm), low-grade nonfunctional PanNETs (NF-PanNETs), which are asymptomatic and sporadic, remains unclear even in consensus guidelines.^5,6,7^ The European Neuroendocrine Tumor Society (ENETS) consensus guidelines suggest surveillance for these lesions.^6^ In the National Comprehensive Cancer Network (NCCN) guidelines, the tumor size recommended for observation as an option has been recently increased from 1.0 cm to 2.0 cm.^5,8^ In contrast, the Japan Neuroendocrine Tumor Society (JNETS) guidelines recommend surgery for all small NF-PanNETs.^7^ The evidence for the treatment selection in patients with subcentimeter NF-PanNETs is lacking due to the rarity of these lesions.^9,10,11,12^

In this study, we evaluated outcomes and treatments of patients with small, localized NF-PanNETs using the National Cancer Database (NCDB). The objective was to evaluate the association between surgical resection for NF-PanNETs measuring 2 cm or smaller and survival.

## Methods

### Data Source

The NCDB is a joint data collection program of the American Cancer Society and the American College of Surgeons Commission on Cancer. The NCDB contains data from approximately 1500 accredited hospitals and clinics and includes approximately 70% of all new cancer diagnoses in the US.^13^ The Colorado Multi-Institutional Review Board deemed this cohort study exempt from review and informed consent requirement because it conducted secondary research on deidentified data and was considered to be non–human participant research according to federal regulations. We followed the Strengthening the Reporting of Observational Studies in Epidemiology (STROBE) reporting guideline.

### Patient Population

We identified patients with PanNENs in the NCDB based on the *International Classification of Diseases for Oncology, Third Edition* (*ICD-O-3*) and *International Statistical Classification of Diseases and Related Health Problems, Tenth Revision, Clinical Modification (ICD-10-CM)* codes (eTable 1 in Supplement 1) between January 1, 2004, and December 31, 2017. Data analysis was performed in June 2022.

We extracted preoperative and postoperative variables and survival outcomes for all patients: age, sex, facility type, Charlson-Deyo comorbidity index, tumor location, tumor size, clinical lymph node metastasis, synchronous distant metastasis, type of surgery, type of chemotherapy, tumor differentiation, pathological lymph node metastasis, lymphovascular invasion, and overall survival (OS). Data on race and ethnicity (American Indian or Alaska Native, Asian, Black, Hispanic, White, other [including Pacific Islander and not specified], or unknown; eTable 2 in Supplement 1) were collected from the NCDB to evaluate whether racial differences were associated with tumor size at diagnosis. We excluded patients without information on OS, surgery, or tumor size. To evaluate the management of small nonmetastatic NF-PanNETs, we also excluded patients with functional tumors, PanNECs, and synchronous distant metastases. In this study, we defined small NF-PanNETs as those that were 2.0 cm or smaller in diameter according to the current guidelines of the NCCN,^5^ North American Neuroendocrine Tumor Society,^8^ ENETS,^6^ and JNETS.^7^

### Statistical Analysis

We grouped small NF-PanNETs into 2 groups according to their diameter: group 1a with tumor size of 1.0 cm or smaller, and group 1b with tumor size between 1.1 and 2.0 cm. Categorical variables were reported as frequency and percentage, and comparisons were made using χ^2^ or Fisher exact tests. We also summarized the baseline characteristics and survival of NF-PanNETs with sizes from 2.1 to 4.0 cm and larger than 4.0 cm to provide a more comprehensive understanding of these lesions.

We evaluated the OS and median survival time using Kaplan-Meier curves and the log-rank test. Multivariable Cox proportional hazards regression models were used to determine the association between preoperative or postoperative variables and survival. Interaction analysis between surgery and each preoperative variable was also performed, which included an interaction term and other preoperative variables in a multivariable Cox proportional hazards regression model. Two-sided *P* values and 95% CIs were reported, and *P* < .05 was considered to be statistically significant. Statistical analyses were performed by R, version 4.1.1 (R Foundation for Statistical Computing).

## Results

### Patient Demographic and Clinicopathological Characteristics

In total, we identified 26 063 patients with PanNENs. In total, 2308 patients had tumors measuring 1.0 cm or smaller, 5938 had tumors measuring 1.1 to 2.0 cm, 9133 had tumors measuring 2.1 to 4.0 cm, and 8684 had tumors measuring larger than 4.0 cm. The demographic and baseline tumor characteristics of each group are shown in eTable 2 in Supplement 1. Most patients with PanNENs were categorized as being of White (77.4%) race, and there were no differences in race and ethnicity among the 4 patient groups. The rates of high-grade tumors, pathological lymph node metastasis, lymphovascular invasion, and synchronous distant metastasis were high when the tumor was large (eTable 2 in Supplement 1). The histologic distribution differed slightly for PanNENs measuring 1.0 cm or smaller vs 1.1 to 2.0 cm. There were more functional PanNENs (1.8% vs 2.9%) and more small cell– and large cell–type PanNECs (0.5% vs 1.1%) in tumors measuring 1.1 to 2.0 cm. Remarkably, 2.3% of subcentimeter NF-PanNETs had distant metastasis at diagnosis.

**Figure 1.**
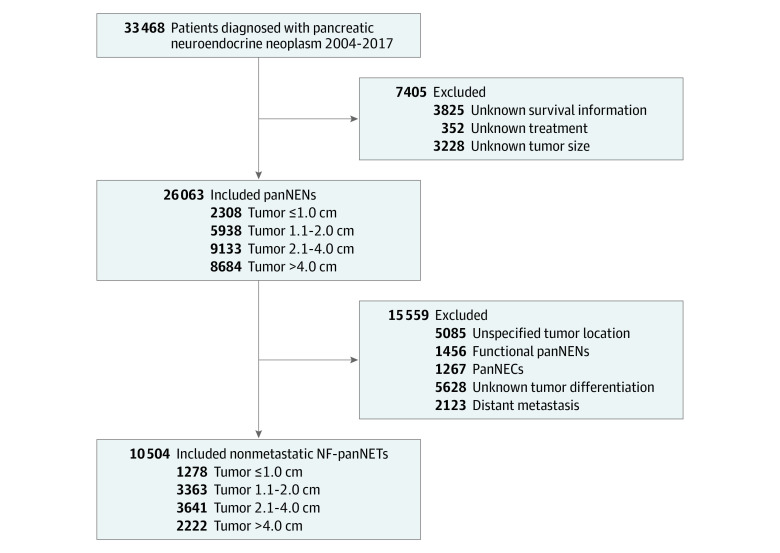
Flowchart of Patients Included in the Study Cohort NF-PanNETs indicates nonfunctional pancreatic neuroendocrine tumors; PanNECs, pancreatic neuroendocrine carcinomas; and PanNENs, pancreatic neuroendocrine neoplasms.

In 10 504 patients who had NF-PanNETs without distant metastasis, there were 1278 patients with tumors measuring 1.0 cm or smaller (group 1a), 3363 with tumors measuring 1.1 to 2.0 cm (group 1b), 3641 with tumors measuring 2.1 to 4.0 cm, and 2222 with tumors measuring larger than 4.0 cm (Figure 1). Of these patients, 4641 were analyzed (1278 in group 1a and 3363 in group 1b). These patients had a mean (SD) age of 60.5 (12.7) years (2338 males [50.4%] and 2303 females [49.6%]) and were mostly White individuals (3593 [77.4%]). The median (IQR) follow-up time was 47.1 (28.2-71.6) months (eTable 3 in Supplement 1).

The surgical resection rate was lower in group 1a than in group 1b (82.0% vs 87.0%; *P* < .001). Table 1 shows the differences in patient characteristics between those with and those without surgical resection. In both group 1a and group 1b, patients with surgical resection were younger than patients without. Tumors located at the pancreas tail more frequently underwent surgical resection in both groups 1a (48.5% vs 30.4%; *P* < .001) and 1b (51.3% vs 32.3%; *P* < .001). In group 1a, more patients without comorbidities underwent observation vs those with comorbidities (177 [77.0%] vs 700 [66.8%]; *P* = .003). In group 1b, patients were more likely to undergo surgical resection at an academic institution or to receive systemic therapy if they did not undergo surgical resection (Table 1). There was no difference in the type of surgery between group 1a and group 1b (eTable 3 in Supplement 1). In group 1b, 285 of 2926 patients (9.7%) had pathological lymph node metastasis and 324 (11.1%) had lymphovascular invasion; even in group 1a, 64 of 1048 patients (6.1%) had pathological lymph node metastasis and 40 (3.8%) had lymphovascular invasion (eTable 3 in Supplement 1). The 90-day postoperative mortality rates for group 1a and group 1b, respectively, were 2.5% and 2.2% for pancreaticoduodenectomy, 1.5% and 0.8% for distal pancreatectomy, and 0% and 3.3% for total pancreatectomy.

**Table 1.** Baseline Characteristics of Patients With Small NF-PanNETs With or Without Surgical Resection by Tumor Size

Characteristic	Patients, No. (%)		*P* value
	With surgical resection (n = 3974)	Without surgical resection (n = 667)	
**Group 1a: tumor size ≤1.0 cm (n = 1278)**			
No. of patients	1048	230	
Age, median (IQR), y	61 (52-69)	65 (57-73)	<.001
Sex			
Male	464 (44.3)	113 (49.1)	.19
Female	584 (55.7)	117 (50.9)	
Facility type			
Nonacademic	224 (21.4)	53 (23.0)	.48
Academic	757 (72.2)	167 (72.6)	
Missing data	67 (6.4)	10 (4.4)	
Charlson-Deyo comorbidity index			
0	700 (66.8)	177 (77.0)	<.001
1	263 (25.1)	28 (12.2)	
2-3	85 (8.1)	25 (10.8)	
Primary site			
Head	255 (24.3)	66 (28.7)	<.001
Body	205 (27.2)	94 (40.9)	
Tail	508 (48.5)	70 (30.4)	
Clinical lymph node metastasis			
Negative	835 (79.7)	216 (93.9)	<.001
Positive	25 (2.4)	2 (0.9)	
Missing data	188 (17.9)	12 (5.2)	
Chemotherapy			
No	1029 (98.2)	224 (97.4)	.23
Single agent	8 (0.8)	2 (0.9)	
Multiple agents	4 (0.3)	2 (0.9)	
Yes (unknown agent)	0	1 (0.4)	
Missing data	7 (0.7)	1 (0.4)	
Histologic distribution			
Islet cell (NOS)	57 (5.4)	5 (2.2)	<.001
Well-differentiated NET	601 (57.3)	175 (76.1)	
Moderately differentiated NET	12 (1.1)	5 (2.2)	
NEN (NOS)	378 (36.0)	45 (19.5)	
Tumor differentiation			
Well	970 (92.6)	211 (91.7)	.68
Moderate	78 (7.4)	19 (8.3)	
**Group 1b: tumor size 1.1-2.0 cm (n = 3363)**			
No. of patients	2926	437	
Age, median (IQR), y	61 (52-69)	69 (60-76)	<.001
Sex			
Male	1520 (51.9)	241 (55.1)	.22
Female	1406 (48.1)	196 (44.9)	
Facility type			
Nonacademic	554 (19.0)	119 (27.2)	<.001
Academic	2157 (73.7)	304 (69.6)	
Missing data	215 (7.3)	14 (3.2)	
Charlson-Deyo comorbidity index			
0	2073 (70.9)	310 (70.9)	.11
1	627 (21.4)	82 (18.8)	
2-3	226 (7.7)	45 (10.3)	
Primary site			
Head	790 (27.0)	175 (40.0)	<.001
Body	634 (21.7)	121 (27.7)	
Tail	1502 (51.3)	141 (32.3)	
Clinical lymph node metastasis			
Negative	2341 (80.0)	404 (92.4)	<.001
Positive	65 (2.2)	9 (2.1)	
Missing data	520 (17.8)	24 (5.5)	
Chemotherapy			
No	2867 (98.0)	411 (94.1)	<.001
Single agent	13 (0.4)	8 (1.8)	
Multiple agents	7 (0.2)	5 (1.1)	
Yes (unknown agent)	1 (0.1)	2 (0.5)	
Missing data	38 (1.3)	11 (2.5)	
Histologic distribution			
Islet cell (NOS)	192 (6.6)	17 (3.9)	<.001
Well-differentiated NET	1628 (55.6)	318 (72.8)	
Moderately differentiated NET	144 (4.9)	11 (2.5)	
NEN (NOS)	962 (32.9)	91 (20.8)	
Tumor differentiation			
Well	2520 (86.1)	385 (88.1)	.30
Moderate	406 (13.9)	52 (11.9)	

Abbreviations: NEN, neuroendocrine neoplasm; NET, neuroendocrine tumor; NF-PanNETs, nonfunctional pancreatic neuroendocrine tumors; NOS, not otherwise specified.

### Survival Analysis in NF-PanNETs 2 cm or Smaller

In all patients with PanNENs, a larger tumor size was associated with worse outcomes (eFigure in Supplement 1). The OS of patients with subcentimeter tumors was better than the OS of patients with tumors measuring 1.1 to 2.0 cm (5-year survival rate, 82.9% vs 81.2%; *P* = .03).

**Figure 2.**
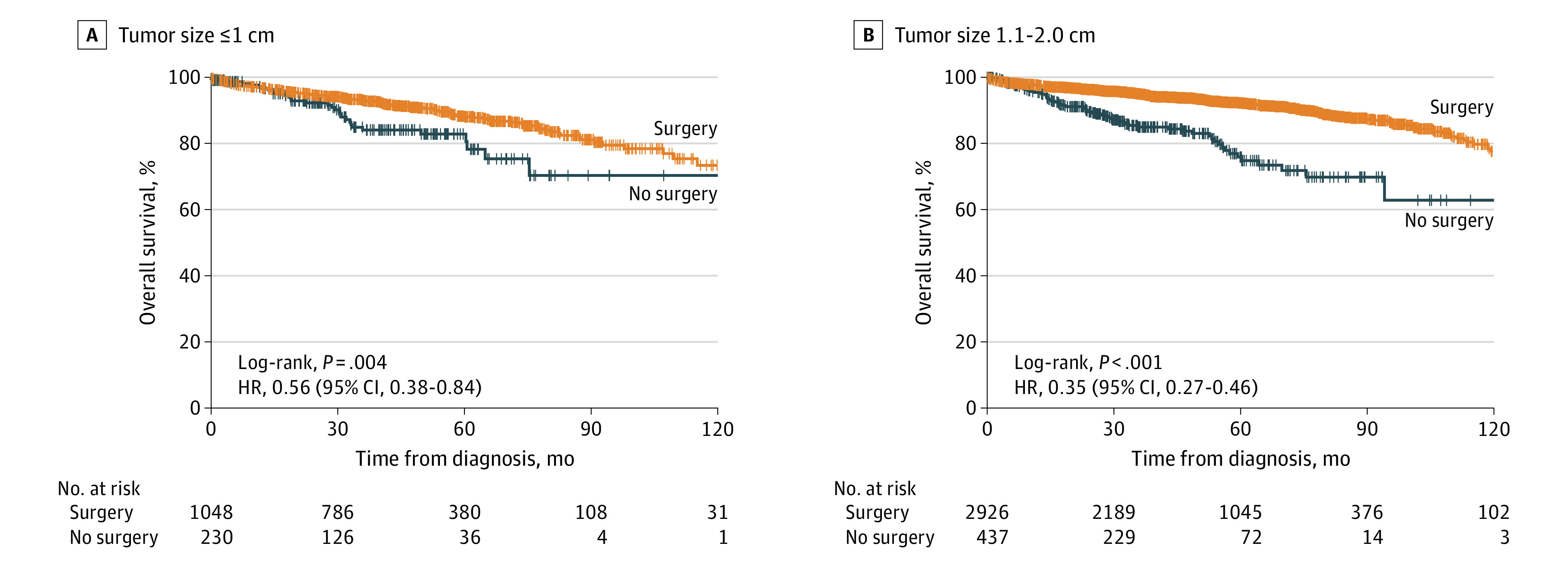
Kaplan-Meier Curves of Overall Survival Stratified by Surgery in Nonfunctional Pancreatic Neuroendocrine Tumors HR indicates hazard ratio.

In patients with small nonmetastatic NF-PanNETs, surgical resection was associated with longer OS in both group 1a (5-year survival rate, 82.8% vs 88.3%; *P* = .004) and group 1b (5-year survival rate, 76.0% vs 92.3%; *P* < .001) (Figure 2A and B). After adjustment for all preoperative covariates, surgical resection was associated with better survival in group 1b (hazard ratio [HR], 0.58; 95% CI, 0.42-0.80; *P* < .001) but not in group 1a (HR, 0.68; 95% CI, 0.41-1.11; *P* = .12) (Table 2). This result was robust even when only well-differentiated NF-PanNETs (*ICD-O-3* code 8240/3) were included (group 1a: HR, 0.74 [95% CI, 0.38-1.42; *P* = .36]; group 1b: HR, 0.60 [95% CI, 0.38-0.93; *P* = .02]). A multivariable Cox proportional hazards regression model showed that in patients with resected small nonmetastatic NF-PanNETs, pathological lymph node metastasis (HR, 1.28; 95% CI, 0.88-1.85; *P* = .43) and lymphovascular invasion (HR, 0.85; 95% CI, 0.55-1.31; *P* = .75) were not associated with OS after adjusting for covariates (eTable 4 in Supplement 1).

**Table 2.** Multivariate Cox Proportional Hazards Regression Models in Small NF-PanNETs

Variable	HR (95% CI)	*P* value
**Group 1a: tumor size ≤1.0 cm (n = 1278)**		
Age, y	1.05 (1.03-1.07)	<.001
Sex		
Male	1 [Reference]	.06
Female	0.68 (0.46-1.01)	
Facility type		
Nonacademic	1 [Reference]	.11
Academic	0.70 (0.45-1.08)	
Charlson-Deyo index		
0	1 [Reference]	.01
1	0.83 (0.51-1.36)	
2-3	2.06 (1.20-3.52)	
Primary site		
Head	1 [Reference]	.78
Body	0.83 (0.49-1.42)	
Tail	0.87 (0.53-1.44)	
Clinical lymph node metastasis		
Negative	1 [Reference]	.99
Positive	1.01 (0.24-4.22)	
Surgery		
No	1 [Reference]	.12
Yes	0.68 (0.41-1.11)	
Chemotherapy		
No	1 [Reference]	.46
Single agent	0.74 (0.10-5.46)	
Multiple agents	0.64 (0.08-5.32)	
Yes (unknown agent)	5.93 (0.57-61.72)	
Tumor differentiation		
Well	1 [Reference]	.27
Moderate	1.45 (0.75-2.81)	
Histologic distribution		
Islet cell (NOS)	1.47 (0.58-3.75)	.60
Well-differentiated NET	1 [Reference]	
Moderately differentiated NET	0.00 (0.00-Infinity)	
NEN (NOS)	1.31 (0.86-2.00)	
**Group 1b: tumor size 1.1-2.0 cm (n = 3363)**		
Age, y	1.05 (1.04-1.07)	<.001
Sex		
Male	1 [Reference]	.28
Female	0.86 (0.66-1.13)	
Facility type		
Nonacademic	1 [Reference]	.01
Academic	0.70 (0.52-0.93)	
Charlson-Deyo index		
0	1 [Reference]	.02
1	1.38 (1.02-1.86)	
2-3	1.61 (1.08-2.41)	
Primary site		
Head	1 [Reference]	.26
Body	0.80 (0.56-1.15)	
Tail	0.78 (0.57-1.07)	
Clinical lymph node metastasis		
Negative	1 [Reference]	.80
Positive	1.10 (0.54-2.26)	
Surgery		
No	1 [Reference]	<.001
Yes	0.58 (0.42-0.80)	
Chemotherapy		
No	1 [Reference]	.001
Single agent	3.83 (1.84-7.99)	
Multiple agents	3.03 (0.95-9.66)	
Yes (unknown agent)	0.00 (0.00-Infinity)	
Tumor differentiation		
Well	1 [Reference]	.42
Moderate	1.20 (0.77-1.86)	
Histologic distribution		
Islet cell (NOS)	1.19 (0.68-2.07)	.45
Well-differentiated NET	1 [Reference]	
Moderately differentiated NET	0.47 (0.16-1.33)	
NEN (NOS)	0.95 (0.71-1.27)	

Abbreviations: HR, hazard ratio; NEN, neuroendocrine neoplasm; NET, neuroendocrine tumor; NF-PanNETs, nonfunctional pancreatic neuroendocrine tumors; NOS, not otherwise specified.

### Interaction Analysis

**Figure 3.**
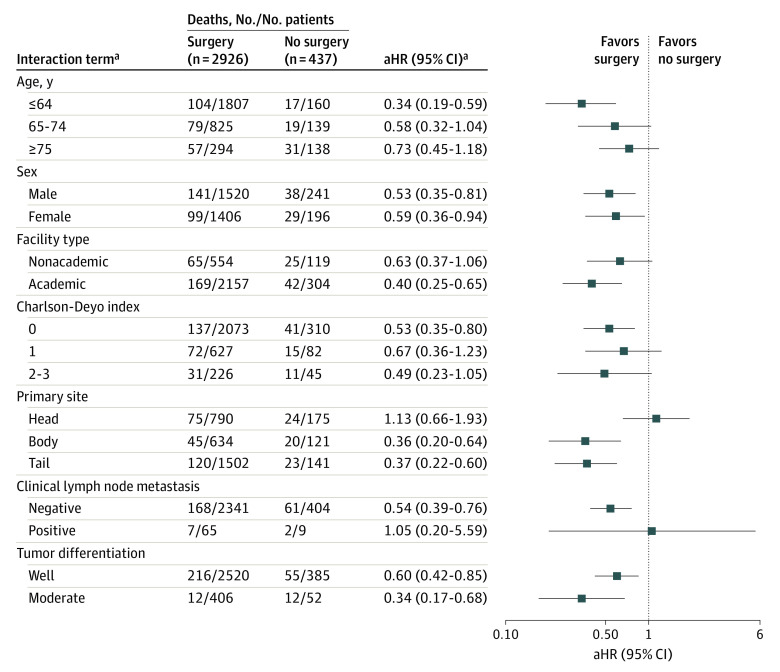
Forest Plot of the Association of Surgery With Mortality in Subgroup Analyses The plot was done on a log scale. The position of each square represents the point estimate of the outcome of surgery, and error bars represent 95% CIs. The vertical line indicates a hazard ratio of 1.0, which was the null hypothesis value. aHR indicates adjusted hazard ratio. ^a^All interactions were tested in 1 model, which was adjusted for age, sex, facility type, Charlson-Deyo index, tumor location, clinical lymph node metastasis, tumor differentiation, tumor histologic distribution, and chemotherapy type. The HR was estimated for each interaction coefficient using the unresected group as the reference.

We analyzed subgroups of patients whom we hypothesized would benefit from surgical resection of NF-PanNETs measuring 1.1 to 2.0 cm (group 1b). Figure 3 summarizes the adjusted HR (aHR) of each interaction term using no surgery as the reference point. Variables that had significant interactions with surgical resection were age 64 years or younger (aHR, 0.34; 95% CI, 0.19-0.59), academic facility type (aHR, 0.40; 95% CI, 0.25-0.65), Charlson-Deyo comorbidity index of 0 (aHR, 0.53; 95% CI, 0.35-0.80), tumor located in the body (aHR, 0.36; 95% CI, 0.20-0.64) or tail (aHR, 0.37; 95% CI, 0.22-0.60), and absence of clinical lymph node metastasis (aHR, 0.54; 95% CI, 0.39-0.76) (Figure 3). Simply stated, the subgroups with these characteristics had a higher survival rate after surgical resection.

## Discussion

In patients with nonmetastatic NF-PanNETs measuring 1.1 to 2.0 cm, surgical resection was associated with better OS (HR, 0.58); however, the finding was not the same for patients with NF-PanNETs 1.0 cm or smaller. Results from the interaction analysis further suggested that patients with NF-PanNETs measuring 1.1 to 2.0 cm who were younger than 65 years, medically uncomplicated, and treated at academic institutions may benefit more from surgical resection. Patients with tumors located at the body or tail of the pancreas may also achieve longer survival with surgery. These data provide support for selecting patients with small nonmetastatic NF-PanNETs for surgical resection.

Although several studies have described the management of small, anatomically resectable NF-PanNETs, their results have been controversial.^9,14,15,16,17,18,19^ To our knowledge, no large study has focused on the management of subcentimeter NF-PanNETs. Some studies reported on the feasibility of observation (instead of surgery) for selected small NF-PanNETs.^9,14,15,16^ Other studies suggested that the potential for malignant transformation might justify the surgical resection of small NF-PanNETs.^17,18,19^ However, the evidence provided by these previous studies was limited due to their small sample size^14^ and lack of survival analysis for subcentimeter NF-PanNETs.^18^ We believe the present study fills those gaps.

Gaujoux et al^20^ examined the natural histories of 38 patients with sporadic, small NF-PanNETs and found that none of the tumors developed distant or nodal metastases after a median (range) follow-up of 34 (24-52) months. Similarly, Sadot et al^14^ found no significant changes in tumor size and no development of metastasis in 104 patients with asymptomatic NF-PanNETs smaller than 3 cm who underwent observation. As a result, the ENETS guidelines recommend a conservative approach for asymptomatic and low-grade small NF-PanNETs.^6,20^ Additionally, the NCCN guidelines for considering observation instead of surgical resection changed in 2021, increasing the maximum tumor size from 1 cm to 2 cm.^5,8,14^ However, given that small NF-PanNETs are generally low-grade tumors, the timeline for observing treatment benefit, or lack thereof, is extremely long.^21,22^ Furthermore, surgeons need to consider the factors associated with short-term and long-term survival in addition to disease-specific survival and to balance the benefits and drawbacks of surgery for this population, such as age, comorbidity status, facility type, and type of surgery. In line with this concept, the present study showed a survival benefit of surgical resection for NF-PanNETs measuring 1.1 to 2.0 cm, which was found specifically for subgroups of patients who had distal pancreatic tumors, were younger than 65 years, had no complications, and were treated at academic centers. These results suggested reasonable criteria for patient selection. However, we did not have a sufficient number of patients to obtain reliable results on the survival benefit of surgical resection for patients with clinical lymph node metastasis.

On the other hand, the rationale for recommending surgery for small NF-PanNETs is their potential malignancy. A large study from the US using the National Cancer Institute SEER (Surveillance, Epidemiology, and End Results) database (1998-2009) showed that, of 263 patients with small NF-PanNENs, 27.3% had pathological lymph node metastasis and 9.1% had distant metastasis.^18^ Ricci et al^17^ performed a systematic review and meta-analysis, including studies involving 382 patients with small NF-PanNENs, and found that the pooled pathological lymph node metastasis rate was 21.2% and the distant metastasis rate was 7.6%. These 2 previous studies concluded that observation for small NF-PanNENs was not safe. However, because the studies included PanNECs and metastatic tumors, the true rate of lymph node and distant metastasis from sporadic, small NF-PanNETs may be lower.^14,23^ Considering this population of small, malignant NF-PanNETs, the JNETS guidelines^7^ recommend surgery for all NF-PanNET cases; however, for selected NF-PanNETs measuring 1.0 cm or smaller, active follow-up may be an option. In the present study, the distant metastasis rate was 2.3% in NF-PanNETs measuring 1.0 cm or smaller and 4.9% in NF-PanNETs measuring 1.1 to 2.0 cm. The lymph node metastasis in nonmetastatic NF-PanNETs was 6.1% in tumors measuring 1.0 cm or smaller and 9.7% in tumors measuring 1.1 to 2.0 cm. In a multivariable Cox proportional hazards regression model, pathological lymph node metastasis and lymphovascular invasion were not factors in outcomes of small nonmetastatic NF-PanNETs. This finding is consistent with previous reports of low-grade NF-PanNETs^11,24^ and may be attributed to small, nonmetastatic NF-PanNETs behaving less aggressively than pancreatic adenocarcinomas when both tumors have lymph node metastasis.^2^ Therefore, even taking into account the low rate of distant metastasis and lymph node metastasis, surgical resection for subcentimeter NF-PanNETs seems overly aggressive.

In recent years, advances in endoscopic ultrasonography–guided fine needle aspiration (EUS-FNA) have played a role in improved preoperative diagnosis, including tumor differentiation and the WHO grade (Ki-67 index).^25^ The diagnostic accuracy of EUS-FNA in evaluating the WHO grading has been reported as 75% to 83%,^26,27,28^ with higher accuracy in smaller tumors due to low tumor heterogeneity.^29,30^ Therefore, we can reliably identify low-grade NF-PanNETs preoperatively and make treatment plans based on their Ki-67 index. In this study, we were not able to investigate the association between the Ki-67 index and OS in small NF-PanNETs because the NCDB lacked data on the Ki-67 index. We expect future studies to evaluate surgical resection for small NF-PanNETs, including the Ki-67 index from EUS-FNA, as a covariate. We believe the results from an international, prospective observational study will provide insight into the management of asymptomatic, sporadic, small NF-PanNENs.^31^

### Limitations

This study has several limitations that are inherent to the use of data from the NCDB. First, coding errors may exist in the database because of the wide variety of *ICD-O-3* histological codes for PanNENs. Second, data on some variables were missing because they were not collected by the NCDB, including the Ki-67 index, symptoms, incidental diagnosis, and recurrence. Therefore, we were unable to define the WHO grade (PanNETs G1, G2, or G3) of each tumor, identify whether the tumor was incidental or asymptomatic, and analyze the disease-free survival. However, because PanNET G3 is rare in small NF-PanNETs, we believe that the implications for survival were minimal. Third, although we performed multivariable analyses, there were still unmeasured confounding variables, such as performance status, that were not included in the NCDB.

## Conclusions

In this nationwide cohort study of small NF-PanNETs, surgical resection of tumors measuring 1.1 to 2.0 cm was associated with prolonged survival in a subgroup of patients who were younger than 65 years, had no comorbidities, received treatment at academic institutions, and had tumors of the distal pancreas. Results of this study support the recommendations of the NCCN guidelines to resect small NF-PanNETs for selected patients. We expect that future studies that include the Ki-67 index will verify these findings.

## Notes

Supplement 1. **eTable 1.** ICD-10 Codes Used for Identifying PanNENs **eTable 2.** Baseline Variables of Patients With PanNENs by Tumor Size **eFigure.** Kaplan-Meier Curves of Overall Survival Stratified by Tumor Sizes of Pancreatic Neuroendocrine Neoplasms **eTable 3.** Baseline Variables of Patients With Nonfunctional PanNETs (cM0) by Tumor Size **eTable 4.** Multivariate Cox Regression Model in Patients With Resected Small Nonfunctional PanNETs (cM0)

Supplement 2.
Data Sharing Statement
