# Supplementary material for: Evaluation of Survival Following Surgical Resection for Small Nonfunctional Pancreatic Neuroendocrine Tumors
Source: JAMA Netw Open. 2023 Mar 28;6(3):e234096. doi: 10.1001/jamanetworkopen.2023.4096 (PMC10051047; doi:10.1001/jamanetworkopen.2023.4096)
Supplement: Supplement 1. — eTable 1. ICD-10 Codes Used for Identifying PanNENs eTable 2. Baseline Variables of Patients With PanNENs by Tumor Size eFigure. Kaplan-Meier Curves of Overall Survival Stratified by Tumor Sizes of Pancreatic Neuroendocrine Neoplasms eTable 3. Baseline Variables of Patients With Nonfunctional PanNETs (cM0) by Tumor Size eTable 4. Multivariate Cox Regression Model in Patients With Resected Small Nonfunctional PanNETs (cM0) [file jamanetwopen-e234096-s001.pdf]

Supplemental Online Content

Sugawara T, Rodriguez Franco S, Kirsh MJ, et al. Evaluation of survival following surgical resection for small nonfunctional pancreatic neuroendocrine tumors. *JAMA Netw Open*. 2023;6(3):e234096. doi:10.1001/jamanetworkopen.2023.4096

eTable 1. ICD-10 Codes Used for Identifying PanNENs

eTable 2. Baseline Variables of Patients With PanNENs by Tumor Size

eFigure. Kaplan-Meier Curves of Overall Survival Stratified by Tumor Sizes of Pancreatic Neuroendocrine Neoplasms

eTable 3. Baseline Variables of Patients With Nonfunctional PanNETs (cM0) by Tumor Size

eTable 4. Multivariate Cox Regression Model in Patients With Resected Small Nonfunctional PanNETs (cM0)

This supplemental material has been provided by the authors to give readers additional information about their work.

© 2023 Sugawara T et al. *JAMA Network Open.*

eTable 1. ICD-10 Codes Used for Identifying PanNENs

ICD-10 code	Guide
ICD-10-CM	
C25.0	Malignant neoplasm of head of pancreas
C25.1	Malignant neoplasm of body of pancreas
C25.2	Malignant neoplasm of tail of pancreas
C25.3	Malignant neoplasm of pancreatic duct
C25.4	Malignant neoplasm of endocrine pancreas
C25.7	Malignant neoplasm of other parts of pancreas
C25.8	Malignant neoplasm of overlapping sites of pancreas
C25.9	Malignant neoplasm of pancreas, unspecified
ICD-O 3rd edition	
8013	Large cell neuroendocrine carcinoma
8041	Small cell neuroendocrine carcinoma
8150	Islet cell tumor (NOS)
8151	Beta cell tumor (insulinoma)
8152	Alpha cell tumor (glucagonoma)
8153	Gastrin cell tumor (gastrinoma)
8154	Mixed endo/exocrine tumor
8155	VIPoma
8156	Somatistatinoma
8158	Endocrine tumor NOS (ACTHoma)
8240	Low grade (well-differentiated) neuroendocrine tumor
8244	Adenoneuroendocrine carcinoma, mixed
8246	Neuroendocrine tumor (NOS)
8249	Moderate differentiated neuroendocrine tumor
8360	Endocrine adenoma
8574	Adenocarcinoma with neuroendocrine differentiation

Abbreviations: ICD, international Classification of Diseases; PanNENs, pancreatic neuroendocrine neoplasms; CM; clinical modification; ICD-O, ICD for oncology; NOS, not otherwise specified.

© 2023 Sugawara T et al. *JAMA Network Open.*

eTable 2. Baseline Variables of Patients with PanNENs by Tumor size

Characteristic	Tumor size, No. (%) ≤ 1.0cm (n = 2308)	1.1 – 2.0 cm (n = 5938)	2.1 – 4.0 cm (n = 9133)	> 4.0 cm (n = 8684)
Median age (IQR) (years)	62 (53 – 70)	62 (52 – 70)	62 (52 – 71)	61 (51 – 70)
Sex				
Male	1030 (45)	3045 (51)	5026 (55)	5026 (58)
Female	1278 (55)	2893 (49)	4107 (45)	3658 (42)
Race/Ethnicity				
White	1758 (76)	4634 (78)	7114 (78)	6659 (77)
Black	286 (12)	676 (11)	1047 (11)	1065 (12)
Hispanic	128 (6)	288 (5)	533 (6)	531 (6)
Asian	86 (4)	206 (4)	250 (3)	259 (3)
American Indian or Alaska Native	5 (0)	17 (0)	30 (0)	28 (0)
Other[a]	23 (1)	51 (1)	67 (1)	58 (1)
Unknown	22 (1)	66 (1)	92 (1)	84 (1)
Primary site				
Head	497 (22)	1581 (27)	3298 (36)	2768 (32)
Body	538 (23)	1080 (18)	1371 (15)	998 (12)
Tail	777 (34)	2211 (37)	2933 (32)	2926 (34)
Other[b]	496 (21)	1066 (18)	1531 (17)	1992 (22)
Synchronous metastasis				
No	2171 (94)	5241 (88)	6042 (66)	4377 (50)
Yes	128 (6)	678 (11)	3069 (34)	4287 (49)
Missing	9 (0)	19 (0)	22 (0)	20 (0)
Surgery				
No	733 (31)	1701 (29)	3671 (40)	4446 (51)
Enucleation	178 (8)	393 (6)	205 (2)	88 (1)
Resection	1407 (61)	3844 (65)	5257 (58)	4150 (48)
Chemotherapy				
No	2196 (95)	5454 (92)	7038 (77)	5582 (64)
Single agent	28 (1)	136 (2)	523 (6)	773 (9)
Multiple agents	46 (2)	206 (4)	1207 (13)	1871 (22)
Yes (Unknown)	5 (0)	26 (0)	109 (1)	198 (2)
Missing	33 (1)	116 (2)	256 (3)	260 (3)
Pathological lymph node metastasis[c]				
Negative	1009 (67)	2647 (62)	2902 (53)	1883 (44)

© 2023 Sugawara T et al. *JAMA Network Open.*

Positive	122 (4)	537 (13)	1855 (34)	1973 (47)
Missing	454 (29)	1053 (25)	705 (13)	382 (9)

	**Tumor size, No. (%)**			
**Characteristic**	**≤ 1.0cm (n = 2308)**	**1.1 – 2.0 cm (n = 5938)**	**2.1 – 4.0 cm (n = 9133)**	**> 4.0 cm (n = 8684)**
Lymphovascular Invasion[c]				
No	1066 (67)	2745 (65)	2402 (44)	1195 (28)
Yes	62 (4)	487 (11)	1352 (25)	1454 (34)
Missing	457 (29)	1005 (24)	1708 (31)	1589 (38)
Margin status[c]				
R0	1427 (90)	3844 (91)	4777 (88)	3465 (82)
R1	65 (4)	160 (4)	315 (6)	328 (8)
R2	3 (0)	6 (0)	21 (0)	46 (1)
Positive (NOS)	29 (2)	101 (2)	175 (3)	252 (6)
Missing	61 (4)	126 (3)	174 (3)	147 (3)
Histology				
Large cell NEC	2 (0)	20 (0)	103 (1)	154 (2)
Small cell NEC	9 (0)	47 (1)	247 (3)	334 (4)
Islet cell (NOS)	145 (6)	441 (7)	644 (7)	697 (8)
Insulinoma	22 (1)	78 (1)	54 (1)	23 (0)
Glucagonoma	4 (0)	11 (0)	22 (0)	28 (0)
Gastrinoma	7 (0)	26 (0)	50 (1)	34 (0)
Mixed endo/exocrine tumor	8 (0)	56 (1)	168 (2)	174 (2)
Vipoma	0 (1)	2 (1)	3 (0)	12 (0)
Somatistatinoma	1 (0)	2 (0)	5 (0)	1 (0)
Well differentiated NET	1214 (53)	2833 (48)	2861 (31)	1937 (22)
NEN (NOS)	854 (37)	2170 (37)	4385 (48)	4739 (55)
Moderate differentiated NET	41 (2)	235 (4)	522 (6)	479 (6)
Adenocarcinoma with neuroendocrine differentiation	1 (0)	17 (0)	69 (1)	72 (1)
Tumor differentiation				
Well	1534 (66)	3746 (63)	4061 (45)	3084 (36)
Moderately	129 (6)	626 (10)	1278 (14)	1334 (15)
Poorly	17 (1)	148 (3)	680 (7)	879 (10)
Undifferentiated	4 (0)	28 (1)	166 (2)	242 (3)
Unknown	624 (27)	1398 (23)	2948 (32)	3145 (36)

© 2023 Sugawara T et al. *JAMA Network Open.*

Abbreviations: PanNENs, pancreatic neuroendocrine neoplasms; IQR, interquartile range; NEC, neuroendocrine carcinoma; NOS, not otherwise specified; NET, neuroendocrine tumor; NEN, neuroendocrine neoplasm.

[a]Other includes Pacific Islander and NOS (not otherwise specified).

[b]Malignant neoplasms with ICD-10-CM codes as C25.3/C25.4/C25.7/C25.8/C25.9.

[c]Patients who underwent surgery.

© 2023 Sugawara T et al. *JAMA Network Open.*

eFigure. Kaplan-Meier curves of overall survival stratified by tumor sizes of pancreatic neuroendocrine neoplasms.

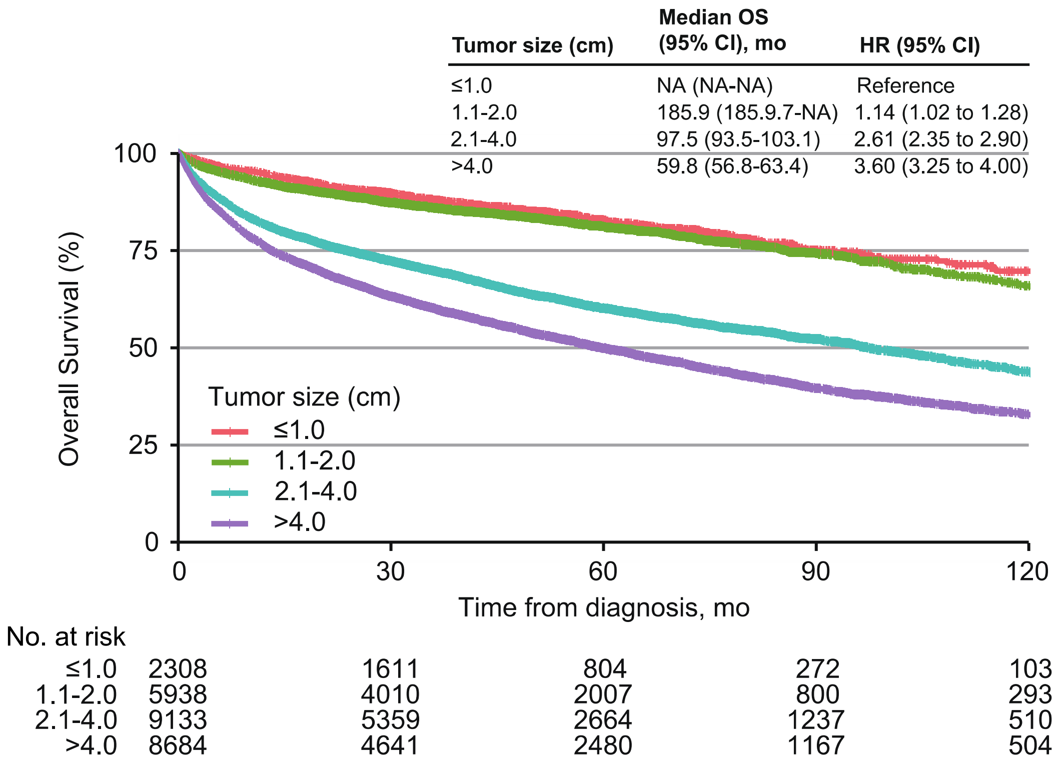

© 2023 Sugawara T et al. *JAMA Network Open.*

eTable 3. Baseline Variables of Patients with Nonfunctional PanNETs (cM0) by Tumor size

	Tumor size, No. (%)			
Characteristic	≤ 1.0cm (n = 1278)	1.1 – 2.0 cm (n = 3363)	2.1 – 4.0 cm (n = 3641)	> 4.0 cm (n = 2222)
Median age (IQR) (years)	62 (53 – 69)	62 (52 – 70)	61 (51 – 69)	61 (51 – 69)
Sex				
Male	577 (45)	1761 (52)	1996 (55)	1184 (53)
Female	701 (55)	1602 (48)	1645 (45)	1038 (47)
Race/Ethnicity				
White	981 (77)	2612 (78)	2800 (77)	1677 (75)
Black	154 (12)	362 (11)	414 (11)	299 (13)
Hispanic	67 (5)	173 (5)	235 (7)	143 (6)
Asian	54 (4)	137 (4)	120 (3)	60 (3)
American Indian or Alaska Native	2 (0)	12 (0)	10 (0)	10 (1)
Other[a]	12 (1)	33 (1)	28 (1)	13 (1)
Unknown	8 (1)	34 (1)	34 (1)	20 (1)
Primary site				
Head	321 (25)	965 (29)	1356 (37)	858 (39)
Body	379 (30)	755 (22)	692 (19)	323 (14)
Tail	578 (45)	1643 (49)	1593 (44)	1041 (47)
Surgery				
No	230 (18)	437 (13)	325 (9)	280 (13)
Enucleation	103 (8)	245 (7)	115 (3)	29 (1)
Pancreatoduodenectomy	282 (22)	828 (25)	1251 (34)	758 (34)
Distal pancreatectomy	581 (45)	1610 (48)	1594 (44)	928 (42)
Total pancreatectomy	60 (5)	181 (5)	265 (7)	165 (7)
Pancreatectomy (NOS)	22 (2)	62 (2)	91 (3)	62 (3)
Chemotherapy				
No	1253 (98)	3278 (97)	3462 (95)	1978 (89)
Single agent	10 (1)	21 (1)	52 (2)	67 (3)
Multiple agents	6 (0)	12 (0)	40 (1)	113 (5)
Yes (Unknown)	1 (0)	3 (0)	10 (0)	10 (1)
Missing	8 (1)	49 (2)	77 (2)	54 (2)
Pathological lymph node metastasis[b]				
Negative	695 (66)	1961 (67)	2017 (61)	1036 (53)
Positive	64 (6)	285 (10)	880 (27)	752 (39)

© 2023 Sugawara T et al. *JAMA Network Open.*

	Tumor size, No. (%)			
Missing	289 (28)	680 (23)	419 (12)	154 (8)
Characteristic	≤ 1.0cm (n = 1278)	1.1 – 2.0 cm (n = 3363)	2.1 – 4.0 cm (n = 3641)	> 4.0 cm (n = 2222)
Lymphovascular Invasion[b]				
No	781 (74)	2106 (72)	1770 (54)	739 (38)
Yes	40 (4)	324 (11)	803 (24)	687 (35)
Missing	227 (22)	496 (17)	736 (22)	520 (27)
Margin status[b]				
R0	959 (92)	2718 (93)	2990 (90)	1713 (88)
R1	40 (4)	91 (3)	165 (5)	105 (5)
R2	2 (0)	2 (0)	4 (0)	11 (1)
Positive (NOS)	15 (1)	54 (2)	74 (2)	73 (4)
Missing	32 (3)	61 (2)	83 (3)	40 (2)
Histology				
Islet cell (NOS)	62 (5)	209 (6)	232 (7)	173 (8)
Well differentiated NET	776 (61)	1946 (58)	1760 (48)	873 (39)
Moderate differentiated NET	17 (1)	155 (5)	301 (8)	213 (10)
NEN (NOS)	423 (33)	1053 (31)	1348 (37)	963 (43)
Tumor differentiation				
Well	1181 (92)	2905 (86)	2842 (78)	1605 (72)
Moderately	97 (8)	458 (14)	799 (22)	617 (28)

Abbreviations: IQR,interquartile range; PanNETs, pancreatic neuroendocrine tumors; NOS, not otherwise specified; NET, neuroendocrine tumor; NEN, neuroendocrine neoplasm.

[a]Other includes Pacific Islander and NOS (not otherwise specified).

[b]Patients who underwent surgery.

© 2023 Sugawara T et al. *JAMA Network Open.*

eTable 4. Multivariate Cox Regression Model in Patients with Resected Small Nonfunctional PanNETs (cM0)

Variable	HR (95% CI)	*P* value
Age (years)	1.05 (1.04-1.06)	<.001
Sex		.03
Male	1 [Reference]	
Female	0.79 (0.64-0.97)	
Facility		.06
Non-academic	1 [Reference]	
Academic	0.75 (0.59-0.95)	
Missing	0.75 (0.30-1.85)	
Charlson-Deyo Score		<.001
0	1 [Reference]	
1	1.23 (0.96-1.56)	
2-3	1.96 (1.42-2.70)	
Primary site		.06
Head	1 [Reference]	
Body	0.70 (0.51-0.95)	
Tail	0.80 (0.62-1.02)	
Pathological T stage[a]		.32
pT0	1.26 (0.17-9.32)	
pT1	1 [Reference]	
pT2	0.66 (0.16-2.68)	
pT3	1.15 (0.77-1.70)	
pT4	0.74 (0.10-5.45)	
Missing	1.49 (1.05-2.12)	
Pathological lymph node metastasis		.43
Negative	1 [Reference]	
Positive	1.28 (0.88-1.85)	
Missing	1.03 (0.80-1.33)	
Lymphovascular invasion		.75
No	1 [Reference]	
Yes	0.85 (0.55-1.31)	
Missing	1.01 (0.75-1.36)	
Margin status		.33
R0	1 [Reference]	
R1	1.39 (0.82-2.36)	

© 2023 Sugawara T et al. *JAMA Network Open.*

R2	4.35 (0.60-31.80)	
Positive (NOS)	1.06 (0.43-2.61)	
Missing	1.44 (0.77-2.69)	

**Variable**	**HR (95% CI)**	***P* value**
Histology		.13
Islet cell (NOS)	0.87 (0.57-1.34)	
Well differentiated NET	1 [Reference]	
Moderate differentiated NET	0.20 (0.05-0.82)	
NEN (NOS)	1.02 (0.81-1.28)	
Tumor differentiation		.81
Well	1 [Reference]	
Moderately	1.05 (0.72-1.53)	

Abbreviations: PanNETs, pancreatic neuroendocrine tumors; NOS, not otherwise specified.

[a] American Joint Committee on Cancer 6th and 7th ed.

© 2023 Sugawara T et al. *JAMA Network Open.*
