# Supplementary material for: Evaluation of Survival Following Surgical Resection for Small Nonfunctional Pancreatic Neuroendocrine Tumors
Source: JAMA Netw Open. 2023 Mar 28;6(3):e234096. doi: 10.1001/jamanetworkopen.2023.4096 (PMC10051047; doi:10.1001/jamanetworkopen.2023.4096)
Supplement: Supplement 2. — Data Sharing Statement [file jamanetwopen-e234096-s002.pdf]

Data Sharing Statement

Sugawara. Evaluation of Survival Following Surgical Resection for Small Nonfunctional Pancreatic Neuroendocrine Tumors. *JAMA Netw Open*. Published March 28, 2023. doi:10.1001/jamanetworkopen.2023.4096

Data
Data available: No
